# The effects of mobile technology-based support on young women with depressive symptoms: A block randomized controlled trial

**DOI:** 10.1097/MD.0000000000036748

**Published:** 2024-01-05

**Authors:** Sookyung Jeong, Chiyoung Cha, Sujin Nam, Jiyoon Song

**Affiliations:** a Department of Nursing, College of Medicine, Wonkwang University, Iksan City, South Korea; b College of Nursing, Ewha Womans University, Seoul City, South Korea; c The University of Honkong, Pokfulam, Hong Kong.

**Keywords:** depressive symptoms, emotional support, female university students, physical activity, wearable device

## Abstract

**Background:**

The current body of knowledge highlights the potential role of mobile technology as a medium to deliver support for psychological and physical health. This study evaluated the influence of mobile technology support on depressive symptoms and physical activity in female university students.

**Methods:**

A block randomized controlled trial design with a single site was used. Ninety-nine participants were block-randomized into 3 arms: Experimental Group 1 (emotional and informational support group), Experimental Group 2 (informational support group), and the control group. Interventions were delivered via mobile technology for 2 weeks. Data on depressive symptoms and physical activity were collected from 84 participants at baseline and on Days 8 and 15. Data analyses included descriptive statistics, *t* tests, one-way analysis of variance, and repeated-measures analysis of variance.

**Results:**

This study showed no interaction effect of time and group on depressive symptom scores and physical activity, considering the emotional and informational support from mobile technology. However, Experimental Group 1 exhibited a significant reduction in depressive symptoms during the first week of the study compared to Experimental Group 2 and the control group. While physical activity in Experimental Group 2 and control group increased only during the first week of the study and subsequently decreased, Experimental Group 1 showed an initial increase during the first week that was sustained into the second week.

**Conclusions:**

Since informational and emotional support showed a strong effect over a short period of time, mobile technology offering emotional support could be used to provide crisis interventions for depression among young women when a short-term impact is required.

## 1. Introduction

### 1.1. Background

Despite global efforts to fight depression, the burden of depression has been increasing, and the treatment gap for depression has not closed^[1]^ especially in young women.^[2]^ reported that female sex was positively correlated with the 12-month prevalence of major depressive episodes in 19 colleges across 8 countries in the United States, Europe, Asia, and Africa. However, college students with mental disorders in 21 countries reported that only 16.5% of them received healthcare.^[3]^ This global trend is also true in South Korea, where women in their twenties reported the highest level of depressive symptoms among all age groups^[4]^ and showed the fewest healthcare visits for depressive symptoms.^[5]^

In addition to gender, research has shown that various social factors influence physical activity and depression in young women. Low levels of self-efficacy regarding physical activity and social support can be a barrier to physical activity among young women with depressive symptoms.^[6]^ In particular, family environment plays an important role in improving physical activity. During childhood, parents and siblings are often inactive and tend to avoid physical activity and sports. Friendship groups also influence young people’s physical activity.^[7]^ When a group is active and pursues sports, they easily engage in the activity, which helps them socialize with friends. These studies clearly showed that positive physical activity experiences in young women are closely correlated with low levels of depression.

For young individuals with unmanaged depression, physical activity can be an effective intervention.^[8–10]^ Physical activity produces an antidepressant effect via both physical and psychosocial pathways.^[11]^ Physically, the hypothalamic-pituitary-adrenal axis responds differently to alleviate depressive symptoms.^[12]^ Physical activity positively affects the hypothalamic-pituitary-adrenal axis over time and helps regulate it by reducing sympathetic nervous system activity, which increases parasympathetic nervous system activity and helps to relax the body.^[13]^ Consequently, physical activity can lead to reduced levels of cortisol and other stress-related hormones^[14]^ and has beneficial effects on depressive symptoms. Physical activity increases the production of endorphins, which are natural mood-boosting compounds.^[15]^ It also increases serotonin levels, which improve mood and reduce anxiety.^[16]^ Consequently, people who engage in physical activity tend to have an increased ability to self-regulate negative emotions, which acts as a buffer against the development of negative emotions.^[17]^

Engaging in and maintaining physical activity is difficult in young women with depressive symptoms. Individuals with depression tend to have sedentary lifestyles.^[18]^ One way to encourage physical activity in this age group is by providing support. A strong positive correlation has been documented between support and physical activity among college students.^[19]^ Recently, support provided through smartphone applications and wearable devices has become an effective way to increase physical activity levels.^[20,21]^ Wearable devices can support physical activities^[22]^ and can be used as part of personalized exercise treatment interventions for depression.^[23]^

Different types of support play different roles in alleviating depressive symptoms and physical activity. Emotional support is defined as the provision of concern, care, values, and existing companions.^[24]^ This causes people to have less risk awareness and burden of problem situations, which contributes to decreasing physiological and emotional impacts.^[24]^ Emotional support can be provided online through communicative behaviors to promote coping with emotional distress.^[25]^ Emotional support has been used in several interventions for patients with depression, such as peer motivation,^[26]^ sending text messages,^[27]^ chatrooms,^[28]^ and social media for patients^[29]^ with depression. Emotional support helps individuals experience low levels of depression, which causes them to promptly recover from mental health problems.^[30,31]^ Further, emotional support, including personalized email feedback, walking with a neighborhood group, and online chats with other participants, increased physical activity.^[32–34]^ Emotional support has also been used to decrease health risk behaviors, including physical inactivity.^[35]^

Information support is provided by offering knowledge in the form of advice, referrals, and feedback on actions.^[36]^ Evidence has shown that informational support enhances self-regulation and changes self-awareness, which supports physical activity and leads to improved physical health.^[37,38]^ Informational support is provided through technical assistance through programs, devices, or accessories. Using wearable devices, such as Fitbit, is a good example of obtaining informational support for maintaining physical activities. It is equipped with various functions for activity tracking, including number of steps, activity time, heart rate, climbing stairs, and energy consumption.^[39]^ To obtain immediate informational support through wearable devices or smartphone applications connected to smartphones, people are interested in and motivated to engage in physical activities.^[40]^ Evidence suggests that individuals are more physically active as their daily step counts increase, and they maintain their activity level by setting daily step goals and competing with family or peer groups with similar devices.^[26,41]^

### 1.2. Objective

This study aimed to examine the effects of mobile technology support on depressive symptoms and physical activity among female university students. The specific aims were to examine the effect of support during the intervention period and the differences between the groups at different time points during the intervention.

### 1.3. Study hypotheses

Hypothesis 1: The emotional and informational group (Experimental Group 1) showed increased physical activity compared to the informational group (Experimental Group 2) and control group after 2 weeks.

Hypothesis 2: The emotional and informational group (Experimental Group 1) will have decreased depressive symptoms compared to the informational group (Experimental Group 2) and the control group after 2 weeks.

## 2. Methods

### 2.1. Research design

A block randomized controlled trial design with a single site was employed. As depression was defined as depressed mood and loss of interest in all activities over 2 weeks^[42]^ and previous studies have shown that compliance with wearable devices decreased after 2 weeks^[43,44]^ we designed a 2-week support program.

### 2.2. Participants

**Figure 1.**
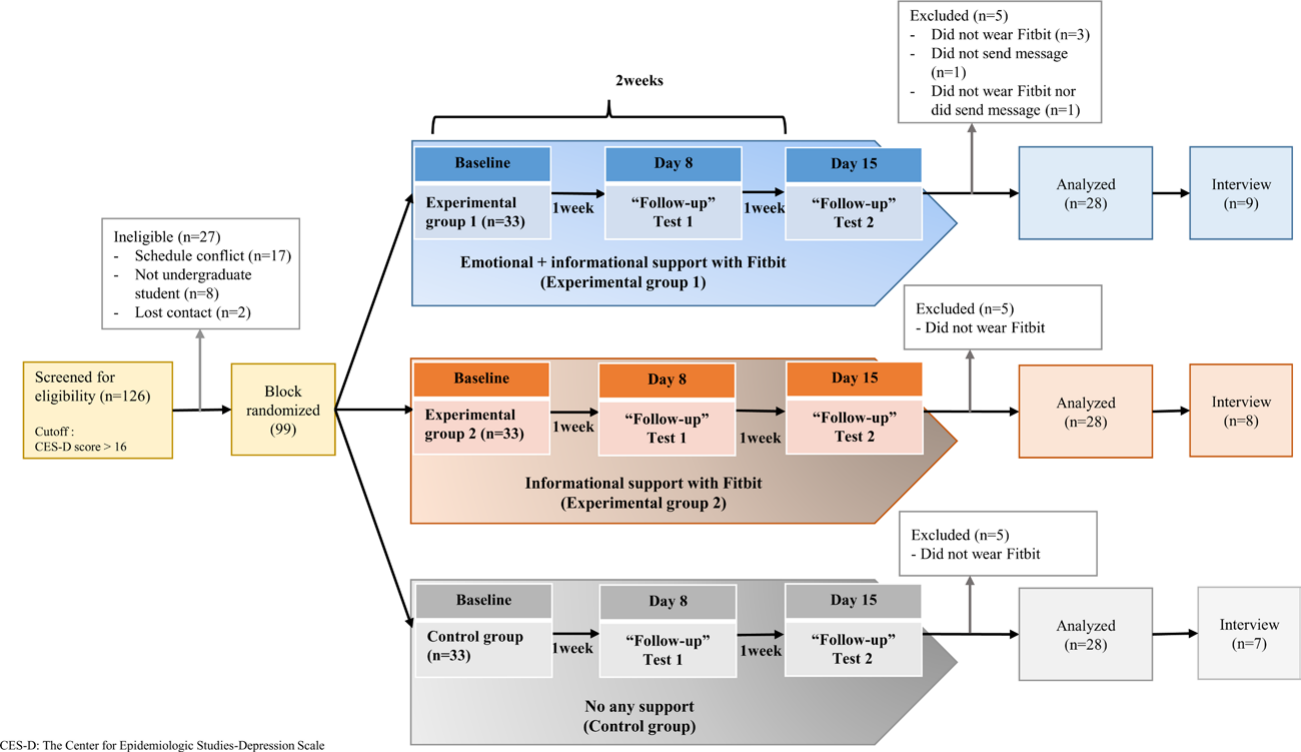
Recruitment process of percipients and intervention. CES-D = The Center for Epidemiologic Studies-Depression Scale.

The participants were female university students who had experienced depressive symptoms in a metropolitan area in South Korea. Flyers for recruitment were posted on a bulletin board at the university campus between April and September 2017. The recruitment process is illustrated in Figure 1. Potential participants downloaded the free research smartphone application “Happy Health 20,” which is a self-screening tool for depressive symptoms based on the Center for Epidemiological Studies Depression (CES-D) Scale.^[45]^ Those who scored >16, the cutoff score for mild depression,^[46]^ contacted the researcher via email. Potential participants were placed on a waitlist until they reached a batch of 24 to 25 potential participants. Using a CES-D score of 16 for mild depression in the South Korean population,^[47]^ block randomization was used to allocate the participants into 3 groups. Once 24 to 25 participants were recruited, their levels of depressive symptoms were categorized into moderate and severe groups based on CES-D score. CES-D scores ranging from 16 to 23 were categorized as moderate, while scores from 24 to 60 were classified as severe.^[47]^ Subsequently, randomized programs (www.randomizer.org) were executed for each group, ensuring that the severity of depressive symptoms was balanced across the 3 groups: Experimental Group 1 (receiving emotional and informational support), Experimental Group 2 (receiving only informational support), and control. The recruitment process was replicated 4 times. Using G*power 3.1, with a medium effect size, alpha (α) level of.05, and power estimate of.8 for a repeated-measures analysis of variance (RMANOVA), at least 72 participants were required. Of 126 screened participants, 99 met the inclusion criteria.

Each group included 33 participants after randomization. After the intervention was completed, we excluded 5 participants from each group because they did not wear the Fitbit for more than a day or had missing data in the questionnaire. Eighty-four participants (28 per group) were included in the final analysis. Interviews were conducted with those who agreed to participate after the intervention, to provide a better understanding of the quantitative results. Twenty-four participants participated in one-on-one interviews with researchers.

Participants were unmarried university students with a mean age of 21.6 ± 3.4 years. No significant differences were found among the 3 groups at baseline (Table 1). Qualitative data were collected to elucidate the influence of the intervention on those who agreed to be interviewed. Twenty-four participants (mean age = 21.17 ± years; range = 18 to 24 years) who agreed to be contacted participated in the interviews.

**Table 1:** Homogeneity test of general characteristics and study variables among the 3 groups.

Variables	Total (N = 84)	EG1 (n = 28)	EG2 (n = 28)	CG (n = 28)	*F (df*)	*χ*^2^ (*df*)	*P* value
Age (yr), mean (SD)	21.6 (3.4)	22.1 (5.5)	21.6 (1.6)	21.0 (1.7)	.7 (*2*)		.48
Perceived economic status, n (%)						13.7 (*8*)	.09
Very low	6 (7.1)	0 (0)	1 (3.6)	5 (17.9)			
Low	30 (35.7)	11 (39.3)	13 (46.4)	6 (21.4)			
Moderate	38 (45.2)	13 (46.4)	13 (46.4)	12 (42.9)			
High	9 (10.7)	4 (14.3)	1 (3.6)	4 (14.3)			
Very high	1 (1.2)	0 (0)	0 (0)	1 (3.6)			
Depressive symptoms, mean (SD)	34.1 (9.0)	31.9 (8.5)	34.2 (8.1)	36.3 (10.1)	1.7 (*2*)		.19
Physical activity, mean (SD)	8210.7 (4989.3)	9484.5 (5658.8)	8276.7 (4920.6)	6870.9 (4091.3)	2.0 (*2*)		.15

CG = control group, EG1 = Experimental Group 1 (emotional and informational support group), EG2 = Experimental Group 2 (informational support group), SD = standard deviation.

### 2.3. Measures

#### 2.3.1. Depressive symptoms.

Depressive symptoms were assessed using the 20-item CES-D scale. Participants were asked about the frequency of symptom occurrence during the previous week on a 4-point Likert-type scale ranging from *rarely* to *most of the time* at baseline (day 0), day 8, and day 15. Potential scores ranged from 0 to 60, with higher scores indicating greater depression. The reliability of research targeting female university students in South Korea using the CES-D was .882 to .920.^[48–50]^ The Cronbach’s alpha for the CES-D was.849 in this study, indicating good internal consistency.

#### 2.3.2. Physical activity.

Fitbit Flex devices (FB 401, Fitbit Inc., San Francisco, CA) were used to track the number of daily steps taken at baseline (day 1), day 8, and day 15. Because the participants started wearing the device on day 0, the daily steps for 24-hour information were gathered starting on day 1. Prior studies have confirmed the reliability and accuracy of the Fitbit Flex.^[51,52]^

### 2.4. Interview

Interviews were conducted in reserved classrooms to protect the participants’ privacy. Open-ended, semi-structured questions were used in the study. Sample interview questions were as follows: “Tell me about your experience of using a wearable device” and “How did getting connected/obtaining information through a wearable device/smartphone application influence you?”

### 2.5. Intervention

The interventions in this study included emotional and/or informational support provided for 2 weeks using a wearable device and smartphone applications (Fig. 1). Table 2 lists the content, frequency, and media used for providing emotional and informational support. After assigning participants to the 3 groups, we explained the research process and intervention to each group in detail.

**Table 2:** Type of emotional and informational support received through a wearable device.

	Emotional support	Informational support	
Contents	- Exchange support messages with group members	-Review depressive symptom scores/daily steps	-Learn strategies to overcome depression and increase daily steps
Frequency	Daily at 8 pm	Daily at 8 pm	Once a week
Medium	Fitbit application	Research application, Fitbit application	Research application

The information commonly provided to Experimental Groups 1 and 2 is as follows. First, they were instructed to download the Fitbit application. Second, we provided the ID and passwords for the Fitbit application, which the participants logged into. Third, the Fitbit device is connected to the administrator’s homepage. We then explained the use of Fitbit to both groups. During the study period, the participants were instructed not to log out of the Fitbit application. Finally, we explained that they would check their CES-D scores weekly by using the research application.

For Experimental Group 1, we explained informational and emotional support. The participants were connected as a group through a Fitbit application to provide emotional support. Emotional support involves sending or receiving supportive messages from a group of 8 to 9 participants to alleviate depressive symptoms and promote physical activity. During orientation, we explained the importance of sending/receiving messages and guided them to send a message at least once every day to all group members, including “Let’s walk hard,” “Cheer up,” and “Fighting.” Even if they could view text messages from their group members in real time, we guided them to check the messages every evening at 8p. m for emotional support.

Each week, the research staff checked whether they had sent messages to all members of the group using the Fitbit homepages. In addition, for information support, we explained that they should review the Fitbit application daily to check the number of steps. In particular, Experimental Group 1 was taught to check the ranking of their daily step counts against those of other group members. Moreover, the research staff explained how to capture and send pictures of their daily steps, and searched for records to overcome depressive symptoms every week.

For Experimental Group 2, only informational support was provided, which included learning strategies to overcome depression, reports of participants’ levels of depressive symptoms, and monitoring of their own physical activities. We explained that they should review the Fitbit application daily in order to check the number of daily steps. They were guided only to check their daily steps, without forming a group. The research staff also explained that they should capture and send pictures of their daily steps and search for records by using research applications to address depressive symptoms.

The control group downloaded the Fitbit application to their smartphones and connected it to a Fitbit band. Researchers logged into the Fitbit homepage and checked their information, then erased the Fitbit application on their smartphones and did not provide the ID and password to prevent them from obtaining their own information on Fitbit. The research staff guided the participants to check their CES-D scores weekly by using the research application. On Day 15, the researchers downloaded their data from the Fitbit homepage.

### 2.6. Data collection

All participants had initial meetings with the researcher to learn about the study, signed an informed consent form, completed the baseline questionnaire, and received guidelines for using Fitbit and smartphone applications for research. As economic status influences depression and health,^[53,54]^ we added this question to the baseline questionnaire.

The participants completed the surveys at baseline and on days 8 and 15. Baseline data collection was performed offline during the initial meeting, and the rest was performed through a smartphone research application. Participants in all 3 groups used Fitbit during the intervention to measure their daily step count.

Interviews were conducted with those who agreed to participate after the intervention, to provide a better understanding of the quantitative results. Interviews were conducted in reserved classrooms to protect the participants’ privacy. The interviews lasted an average of 45.26 minutes in average. All interviews were recorded using digital recorders and transcribed verbatim with participants’ permission.

### 2.7. Data analysis

Analysis of the survey and physical activity data was conducted using IBM SPSS Statistics version 25.0. The homogeneity of the sample at baseline and differences in outcomes at different time points were analyzed using one-way ANOVAs and chi-squared (*χ*^2^) tests. RMANOVA was used to identify the effects of the intervention on outcome variables over time. We applied Greenhouse-Geisser corrections to estimate the variables. The relationship between depression and physical activity was analyzed using Pearson’s correlation coefficient.

### 2.8. Ethical considerations

The Institutional Review Board of Ewha Womans University reviewed and approved this study (IRB number: 109-20). The purpose and procedures of the study were explained in detail to participants using an informed consent form. The identifiable information was coded using participant numbers to ensure anonymity. Referral lists for depression were provided during meetings and smartphone research applications. During the intervention, participants in the control group did not have the opportunity to obtain information about their daily steps while wearing the Fitbit. They had the option of using the Fitbit band and the application for 1 week after the intervention.

## 3. Results

Table 3 reports the differences in the outcomes among the groups over time. Mauchly’s test of sphericity was significant for the analysis of depressive symptoms (*P* < .001) and physical activity (*P* = .01); therefore, multivariate analysis with Greenhouse-Geisser epsilon correction was used. There was a significant difference in depressive symptoms among the groups (*F*[2,810] = 3.03, *P* = .05), which decreased significantly at each time point (*F*[2,162] = 27.83, *P* < .001). All groups showed decreased depressive symptoms on Day 15, and the scores of Experimental Group 1 dropped sharply on Day 8. Their depression scores decreased by 5.79, whereas those of the other 2 groups (Experimental Group 2 and the control group) decreased by 3.78 and 3.54, respectively. After 1 week, the depressive symptoms score of Experimental Group 1 slightly increased by 0.68, and the other 2 groups showed mild decreases of 1.78 and 1.08, respectively. Time and group did not show an interaction effect for the depressive symptoms scores (*F*[4,810] = .65, *P* = .60).

**Table 3:** Differences among study variables over time.

Outcome	Group	Baseline	Day 8	Day 15	Source	*F* test (*df*)	*P* value
		Mean (SD)					
Depressive symptoms	EG1 (n* = *28)	29.43 (8.16)	23.64 (7.03)	24.32 (8.62)	Group	3.03 (*2,810*)	.05
	EG2 (n* = *28)	30.50 (7.34)	26.96 (8.00)	25.18 (8.00)	Time	27.83 (*2,162*)	<.001
	CG (n* = *28)	33.57 (8.66)	29.79 (9.61)	28.71 (10.49)	Time × Group	.65 (4,810)	.60
Physical activity	EG1 (n* = *28)	9484.50 (5658.81)	11,025.99 (3815.10)	11,423.68 (4845.77)	Group	4.72 (*2.810*)	.01
	EG2 (n* = *28)	8276.70 (4920.62)	10,672.21 (5960.70)	8452.54 (5062.31)	Time	2.53 (*13,105*)	.004
	CG (n* = *28)	6870.86 (4091.32)	7151.09 (3376.27)	6735.12 (5091.93)	Time × Group	1.36 (*26,105*)	.13

CG = control group, EG1 = Experimental Group 1 (emotional and informational support group), EG2 = Experimental Group 2 (informational support group), SD = standard deviation.

There were significant differences in physical activity among the 3 groups (*F*[2,810] = 4.72, *P* = .01) and at each time point (*F*[13,105] = 2.53, *P* = .004). Experimental Groups 1 and 2 showed sharp increases in daily steps of 1541.49 and 2395.51, respectively, on Day 8, whereas the score of the control group slightly increased by 280.23. Between Days 8 and 15, the figure for Experimental Group 1 continuously increased by 397.69, that for Experimental Group 2 sharply decreased by 2219.67, and that for the control group decreased by 415.97. Time and group did not show an interaction effect for physical activity (*F*[26,105] = 1.36, *P* = .13). Furthermore, as shown in Table 4, Pearson’s correlation analysis revealed a negative correlation between physical activity and depression; depressive symptoms decreased as physical activity increased.

**Table 4:** Correlation between depression and physical activities.

Variables	Depression *r (p*)	Physical activity *r (p*)
Depression	1.00	−.0136 (.031)
Physical activity	−.0136 (.031)	1.00

## 4. Discussion

This study evaluated the effects of Fitbit in terms of depressive symptoms and physical activity. Experimental group 1, with emotional and informational support, exhibited a rapid decrease in depressive symptoms on Day 8, and physical activity increased and remained elevated until Day 15.

Results of this study showed a negative relationship between the 2 variables. Previous studies have suggested that adults with depressive symptoms exhibit decreased physical activity when measured with wearable devices.^[55]^ Additionally, being physically active once or twice a week decreases the risk of depression.^[56]^ This may be due to the antidepressant effects of physical activity, both physical and psychological.^[11]^ Mobile technology-based devices, including the Fitbit or smart watch, are designed to be easy to wear on the wrist with a lightweight design, and they are convenient to wear continuously in daily life and to record various activities. Additionally, they provide user-friendly applications that trace step count, distance traveled, and the number of stairs climbed in real time.^[39]^ Therefore, many people have recently started using this kind of device to manage not only physical health but also mental health. Some mobile technology-based devices even help to manage depression and anxiety.^[57]^ Young people adapt and learn to use a highly technological, new device more easily compared to older people. Therefore, considering the benefits of using Fitbit and the connection between physical activity and depression, mobile technology-based support to improve physical activity and alleviate depressive symptoms may benefit young women with depression.

The RMANOVA indicated a significant effect on depressive symptoms over time. Participants in Experimental Group 1 experienced dramatic alleviation of depressive symptoms on Day 8. Experimental Group 2 also showed decreased depressive symptoms over time, and the degree of reduction in depression scores was similar between Experimental Groups 1 and 2 after 2 weeks. Notably, it has been shown that when emotional support is added, the effect can become apparent within a short time. This finding is encouraging, considering the large treatment gap for depression among young women. In accordance with this finding, young adults reported that wearable devices, including the Fitbit and smart watch, were useful and acceptable for monitoring depressive symptoms.^[58]^ The intervention used in this study could be a viable method to support the management of depressive symptoms in young women, and many previous studies have warned that the combination of depression and impulsivity can lead to suicide.^[59,60]^ Therefore, emotional and informational support using mobile technology-based wearable devices could be used to control crisis interventions for depression in young women, when a strong effect over a short period is required.

Experimental Group 1 steadily increased their physical activity levels compared with Experimental Group 2 and the control group over the 2-week intervention period. In line with these findings, the short-term use of Fitbit can increase physical activity.^[61,62]^ Although young people may be motivated to engage in physical activity, their compliance rates may decrease over time.^[63]^ People may become less interested in using wearable devices after forming health patterns.^[64]^ Similarly, the long-term use of wearable devices is not guaranteed to increase physical activity levels.^[65]^ In our study, when the 2 groups were compared, Experimental Group 1, which received emotional and informational support, showed a significant increase over 2 weeks. However, Experimental Group 2, with only informational support, showed increased physical activity on Day 8 but not on Day 15. Many studies have verified the importance of social support, including message boards and online chat sessions, in increasing physical activity.^[66]^ These findings indicated that emotional support is an important factor.

Researchers have been interested in smart healthcare systems such as smartphone applications, portable devices, and mobile technology-based devices. These devices are typically used to diagnose diseases and to monitor and manage physiological data.^[67,68]^ Recently, the possibility of using wearable devices as psychological interventions for patients with mental health issues has been proposed.^[69]^ However, few smart healthcare systems that provide integrative care involving both physical and mental health have been developed. This intervention can be further developed into a real-time data-driven support tool for psychological interventions. In addition, the accumulation of data and connections between individuals and health outcomes, such as physiological indices, may make such data more useful. Inter-professional collaboration among healthcare system administrators, big data specialists, and health professionals can help accomplish this goal.

### 4.1. Limitations

This study had some limitations. First, only a relatively small number of participants were included. Qualitative data were used as supplementary data to the quantitative results to validate our findings. Second, several participants were excluded because they did not wear the device daily or because they had missing data. Third, participants used 2 different types of applications. Although the compliance rate was high, it would have been much easier if participants had used only 1 multifunctional smartphone application. In the future, a multifunctional smartphone application tailored to the needs of each individual would be beneficial in providing tailored interventions. Fourth, the total depressive symptom scores in Experimental Groups 1 and 2 decreased after 2 weeks. Studies on the changes in depressive symptoms according to the period of physical activity are recommended to clearly identify the effects of physical activity on depression. Fifth, the control group showed a steady decrease in depressive symptoms during the intervention period. During the experimental period, they wore Fitbit bands without any information, such as bracelets, which led to a decrease in their depressive symptoms. This may reflect a “placebo effect.”^[70,71]^ Although they did not receive any intervention, they experienced feelings of receiving emotional care, which could be a psychological benefit. Additionally, although the research staff did not provide information on research applications related to overcoming depression, the control group members might have read the content, which could eventually influence their mental health. However, further studies are required to evaluate these effects. Sixth, participants were enrolled 3 times during the examination period. Therefore, physical activity may be influenced by these factors, the 2-week intervention period could have been too short to measure meaningful and sustainable changes in physical activity and depressive symptoms. Future research should consider longer intervention periods to better assess the efficacy and generalizability of interventions that target physical activity and depressive symptoms. Finally, the number of steps required for physical activity was measured. This can be a useful tool for monitoring physical activity but can also provide a misleading picture of an individual’s overall activity. Therefore, future research should consider other aspects of physical activity beyond step count and intensity of physical activity to evaluate its effects on depressive symptoms.

### 4.2. Conclusions

Female university students with depressive symptoms received emotional and/or informational support from wearable devices and mobile devices. Providing both emotional and informational support did not have a significant reductive effect on depressive symptoms and physical activity in this study but decreased depressive symptoms and increased physical activity levels compared to baseline. Furthermore, the information group without emotional support did not show a significant difference and only showed a decrease in depressive symptom levels after 2 weeks. Consequently, anonymous emotional support is vital for continued participant compliance with the intervention. However, no significant results were obtained in this study. Therefore, further research using mobile technology should be conducted to evaluate the effects of depressive symptoms on physical activity among young women.

## Author contributions

**Conceptualization:** Sookyung Jeong, Chiyoung Cha.

**Data curation:** Sujin Nam, Jiyoon Song.

**Formal analysis:** Sookyung Jeong, Sujin Nam.

**Funding acquisition:** Chiyoung Cha.

**Methodology:** Chiyoung Cha.

**Supervision:** Chiyoung Cha.

**Validation:** Sookyung Jeong, Sujin Nam, Jiyoon Song.

**Visualization:** Sookyung Jeong.

**Writing – original draft:** Sookyung Jeong, Chiyoung Cha, Sujin Nam.

**Writing – review & editing:** Sookyung Jeong, Chiyoung Cha.

## Correction

The second author’s name was originally published incorrectly. The author’s name was corrected to Chiyoung Cha in the author list and the author contributions section.
